# Safety and Immunogenicity of a Vaccine Against Coxsackieviruses B (PRV-101)—Follow-up of the First-in-Human Phase 1 Trial

**DOI:** 10.1093/ofid/ofag277

**Published:** 2026-05-13

**Authors:** Jutta E Laiho, Jussi P Lehtonen, Leena Puustinen, Susanna Kääriäinen, Taina Härkönen, Sami Oikarinen, Francisco León, Miguel Sanjuan, Mika Scheinin, Mikael Knip, Heikki Hyöty

**Affiliations:** Department of Virology, Faculty of Medicine and Health Technology, Tampere University, Tampere, Finland; Department of Virology, Faculty of Medicine and Health Technology, Tampere University, Tampere, Finland; Department of Virology, Faculty of Medicine and Health Technology, Tampere University, Tampere, Finland; Clinical Research Services Turku—CRST Oy, Turku, Finland; Research Program for Clinical and Molecular Metabolism, Faculty of Medicine, University of Helsinki, Helsinki, Finland; Department of Virology, Faculty of Medicine and Health Technology, Tampere University, Tampere, Finland; Provention Bio, Inc., A Sanofi Company, Bridgewater, New Jersey, USA; Provention Bio, Inc., A Sanofi Company, Bridgewater, New Jersey, USA; Clinical Research Services Turku—CRST Oy, Turku, Finland; Institute of Biomedicine, University of Turku, Turku, Finland; Research Program for Clinical and Molecular Metabolism, Faculty of Medicine, University of Helsinki, Helsinki, Finland; Department of Pediatrics, Tampere University Hospital, Tampere, Finland; Department of Virology, Faculty of Medicine and Health Technology, Tampere University, Tampere, Finland; Department of Pediatrics, Tampere University Hospital, Tampere, Finland; Fimlab Laboratories, Tampere, Finland

**Keywords:** celiac disease, coxsackie B virus, neutralizing antibodies, type 1 diabetes, vaccine

## Abstract

**Background:**

Coxsackie B viruses cause acute infections and have been linked to chronic diseases like cardiomyopathies, type 1 diabetes, and celiac disease. Despite their clinical significance, no vaccines exist for coxsackie B virus types. PRV-101, a new candidate vaccine covering 5 coxsackie B virus types, showed good immunogenicity and tolerability in a phase 1 trial (PROVENT) in adults.

**Methods:**

We conducted an extended follow-up of the PROVENT trial to assess the long-term immune response and safety of PRV-101. A total of 26 participants from the original cohort (n = 32) were enrolled for additional testing ∼2 years postimmunization (11 high-dose, 10 low-dose, and 5 placebo). Coxsackie B virus–specific antibody responses were measured and compared with earlier time points.

**Results:**

PRV-101 was safe, with no late adverse effects or emergence of autoantibodies linked to type 1 diabetes or celiac disease. Neutralizing virus antibodies remained elevated, with a clear dose-dependent response. In the high-dose group, antibodies against all coxsackie B virus types reached presumably protective levels, except for coxsackie B virus 2, where 2 participants turned seronegative. Enzyme-linked immunosorbent assay tests confirmed elevated antibody levels against coxsackie B virus proteins.

**Conclusions:**

These results suggest that PRV-101 induces durable antibody responses lasting for at least 2 years. The findings support the continued development of PRV-101 for preventing both acute coxsackie B virus infections and chronic diseases like type 1 diabetes and celiac disease.

Coxsackievirus B (CVB) infections are common in all age groups. They can cause a variety of symptoms and conditions, ranging from mild common cold–type respiratory disease to severe illnesses, such as meningitis, encephalitis, myocarditis, and hand, foot, and mouth disease [1]. In young infants, severe CVB infections are sometimes fatal. In addition, CVBs have been linked to chronic diseases such as chronic cardiomyopathies, type 1 diabetes, and celiac disease, where persistent CVB infection in the heart, pancreas, and gut mucosa, respectively, may be involved [2, 3]. There are >110 human-infecting enterovirus types that are divided into 4 species, *Enterovirus A–D*. CVBs belong to *Enterovirus B* and include 6 types. The medical significance of these infections is demonstrated by the fact that among the >110 different enterovirus types, CVBs have continuously been among those 15 virus types that have most frequently led to health care contacts in the United States [4, 5]. In spite of the significant disease burden, no vaccine is available for the prevention of CVB-related diseases.

We started a clinical development program aimed at a pentavalent human CVB vaccine capable of protecting against CVB-associated diseases. This formalin-inactivated whole-virus vaccine (PRV-101) includes the most frequent CVB types (CVB1–5) and contains no adjuvant. PRV-101 was recently tested in a first-in-human phase 1 trial (PROVENT). Three immunizations 1 month apart did not lead to vaccine-associated severe adverse effects [6]. Three doses of PRV-101 induced robust dose-dependent antibody responses against each of the 5 CVB types included in the vaccine. The last study visit occurred 24 weeks after the last immunization. At that time, the vaccine-induced antibody levels already showed some decline, still remaining at high levels, exceeding those considered protective against enterovirus infections. The aim of the present study was to evaluate the longer-term safety and immune response of PRV-101 by analyzing the antibody levels at a follow-up visit, ∼2 years after the last immunization. Here we report that PRV-101-induced neutralizing antibody responses persisted throughout this longer observation period at levels that are considered protective against CVB infections.

## METHODS

### Study Design and Trial Participants

This is an investigator-initiated follow-up study (IIS) of safety and long-term immune response in participants of the PROVENT trial, a first-in-human phase 1 trial of PRV-101, a CVB vaccine candidate (hereafter termed PROVENT-IIS), EudraCT number 2022-004174-39, funded by Provention Bio (later acquired by Sanofi). The principal investigator was Professor Heikki Hyöty (Tampere University, Tampere, Finland), and Tampere University, Tampere, Finland, was the study sponsor. Recruitment of study subjects was carried out by Clinical Research Services Turku (CRST Oy; Turku, Finland). Ethical approval was obtained from the National Committee on Medical Research Ethics (TUKIJA), and the trial protocol was approved by the Finnish Medicines Agency (Fimea).

**Figure 1.**
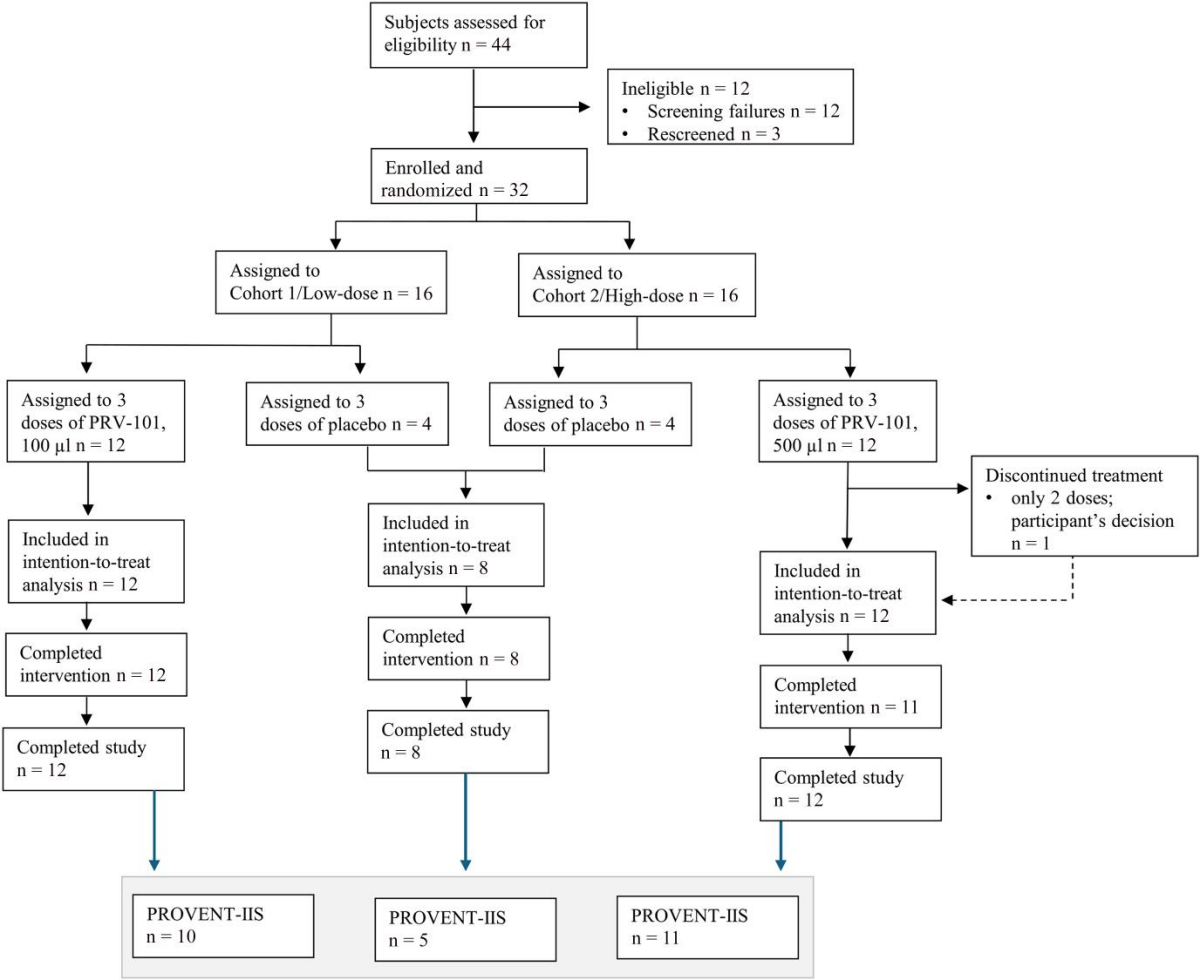
PROVENT trial profile and participants enrolled in PROVENT-IIS from the 3 trial arms.

The recruitment and randomization of the 32 participants in the original PROVENT trial have been described previously [6]. Briefly, participants were randomized into 3 parallel dosing cohorts as follows: low dose (100 µL) PRV-101 (n = 12), high dose (500 µL) PRV-101 (n = 12), and placebo (n = 8). In the present study, we invited all 32 participants to attend an additional study visit (IIS visit) by contacting them by phone. The IIS visit occurred 69–88 weeks after the end-of-study (EOS) visit, which corresponds to 101–120 weeks (mean 108 weeks = 27 months) after the first PRV-101 dose and 93–112 weeks (mean 100 weeks = 23 months) after the last PRV-101 dose. The PROVENT trial profile amended with PROVENT-IIS is shown in Figure 1.

**Figure 2.**
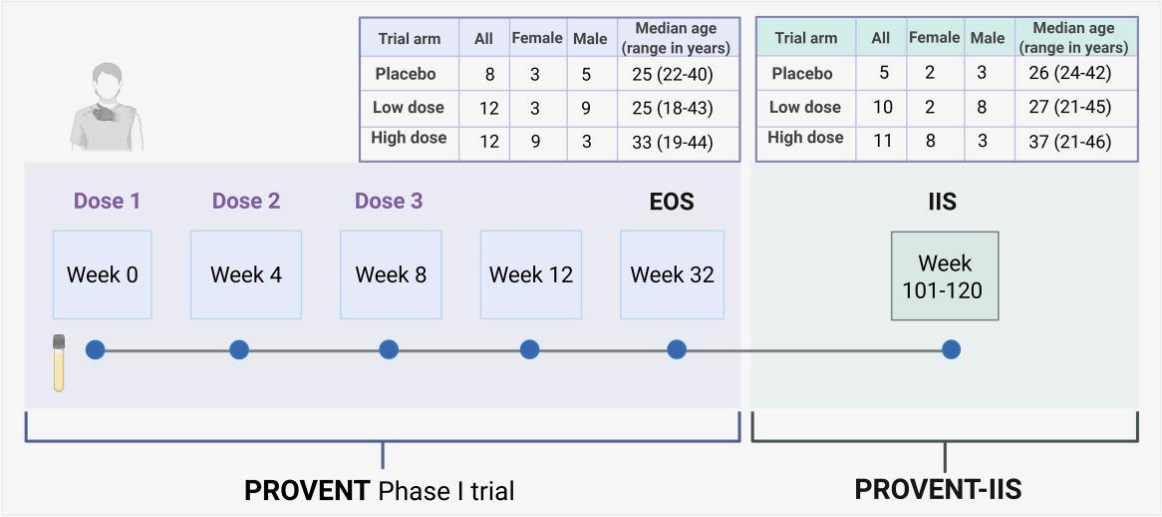
Schematic illustration of the PROVENT trial and PROVENT-IIS designs. In the original PROVENT Phase I trial, participants were given 3 i.m. injections of PRV-101 or placebo at intervals of 4 weeks and followed for safety for 24 weeks until the EOS visit. Blood and nasal swab samples were also collected at each visit. We were able to recruit 26 of the PROVENT participants to PROVENT-IIS. An additional blood sample was collected at the IIS time point, 101 to 120 weeks after the first injection of PRV-101 or placebo. For the PROVENT trial, virus antibody analyses and type 1 diabetes and celiac disease autoantibody analyses were carried out from week 0, 4, 8, 12, and EOS samples and, for PROVENT-IIS, from the EOS and IIS samples. The age of the participants in both studies was calculated at the time of consent for the PROVENT Phase I trial. Created with Biorender.com. Abbreviations: EOS, end of study; IIS, investigator-initiated follow-up study; i.m., intramuscular.

Altogether 26 subjects consented to PROVENT-IIS and were invited to visit the CRST's clinic. The subjects belonged to all 3 trial arms, representing placebo (n = 5), low doses (n = 10), and high doses (n = 11) of PRV-101. At CRST, the health status of the participants was recorded by a questionnaire, and a blood sample was drawn. The serum fraction was used for the analysis of CVB antibodies and for autoantibodies associated with type 1 diabetes or celiac disease. Figure 2 presents a schematic illustration of the study timeline and provides a summary of the age and sex distribution of the study participants.

### Virus Antibodies

We carried out virus antibody analyses from the collected IIS samples of the 26 participants as well as from their corresponding EOS samples, collected in the main PROVENT trial. The analyses were done in the same virus laboratory at Tampere University, using the same assays as in the main PROVENT trial [6]. Briefly, virus type–specific antibodies against each of the CVB1–5 strains included in PRV-101 were analyzed using plaque reduction neutralizing antibody assays. The end-point neutralizing antibody titer was determined by 2-step dilution series of serum samples.

The enzyme-linked immunosorbent assay (ELISA) immunoglobulin (Ig) G and IgM class CVB antibody levels in serum were analyzed using the same commercial antibody assays as in the main PROVENT trial (SERION ELISA classic Coxsackievirus IgG [ESR134G], IgM [ESR134M] Serion GmbH, Würzburg, Germany) according to the manufacturer's instructions with a single serum dilution (IgM 1:100, IgG 1:500). In these tests, the antigen is a mixture of recombinant antigens derived from conserved and subtype-specific epitopes of the VP1 proteins of coxsackieviruses B1, B3, and B5. Antibody units were calculated according to the manufacturer's instructions.

### Autoantibodies

We analyzed the autoantibodies against type 1 diabetes associated islet cell antigens (antibodies to insulin [IAA], truncated GAD65 [GADA], islet antigen 2 [IA-2A], and zinc transporter 8 [ZnT8A]) in the PEDIA laboratory, University of Helsinki, and celiac disease–associated antitransglutaminase IgG and IgA class antibodies in Turku University Hospital, similar to the procedures in the main PROVENT trial [6, 7].

### Statistical Analyses

Virus-neutralizing antibodies and autoantibodies were analyzed both from the IIS time point and from the EOS sample of the main PROVENT trial. This enabled the adjustment of the virus antibody levels detected in the samples collected in the current study (weeks 101–120 after baseline) to the levels detected in the original analyses of the EOS samples. The adjustment, referred to as “EOS-adjusted” hereafter, was done using the following formula:


$$\begin{array}{rl} \mathrm{EOS}-\mathrm{adjusted}\;{\mathrm{titer}}_{101-120}= & ({\mathrm{Titer}}_{32}/{\mathrm{Titer}}_{32\;\mathrm{IIS}\;\mathrm{repeat}}{)}^{*}\\ & {\mathrm{Titer}}_{101-120}, \end{array}$$

where the numbers indicate the weeks after the first dose of vaccine, with EOS time point being week 32 and IIS time point being weeks 101–120. These adjusted antibody levels were used when the overall kinetics of antibody levels were analyzed from the baseline sample and all following samples, including the IIS follow-up sample.

Antibody levels were log-transformed to present the variation in the vaccine-induced antibody responses, log2-transformed for virus neutralizing titers, and ln-transformed for ELISA antibodies. Mean and median values of the log-transformed antibody levels were used in representative figures. A linear mixed-effects model with participant as a random intercept was used to analyze differences between study arms at different time points. As the primary aim of this trial was to evaluate the safety and tolerability of PRV-101, it was not designed to test statistical inferences about immunogenicity; the immunogenicity assessments were exploratory in nature. Therefore, we did not correct the *P* values for multiple comparisons. R, version 4.2.1 (https://www.r-project.org/, accessed June 23, 2023), was used for statistical analysis.

## RESULTS

### Health Status of Trial Participants

The health status of all trial participants was evaluated by a questionnaire at CRST during the IIS study visit. None of them had developed type 1 diabetes, celiac disease, or any other chronic disease after the EOS visit of the main PROVENT trial. No new chronic medications had been started either.

### Autoantibodies

All IIS participants remained negative for all tested type 1 diabetes–related islet autoantibodies (IAA, GADA, IA-2A, ZnT8A) and celiac disease–related IgG and IgA class antitransglutaminase autoantibodies.

### Neutralizing Antibodies Against Different Coxsackievirus B Types

**Figure 3.**
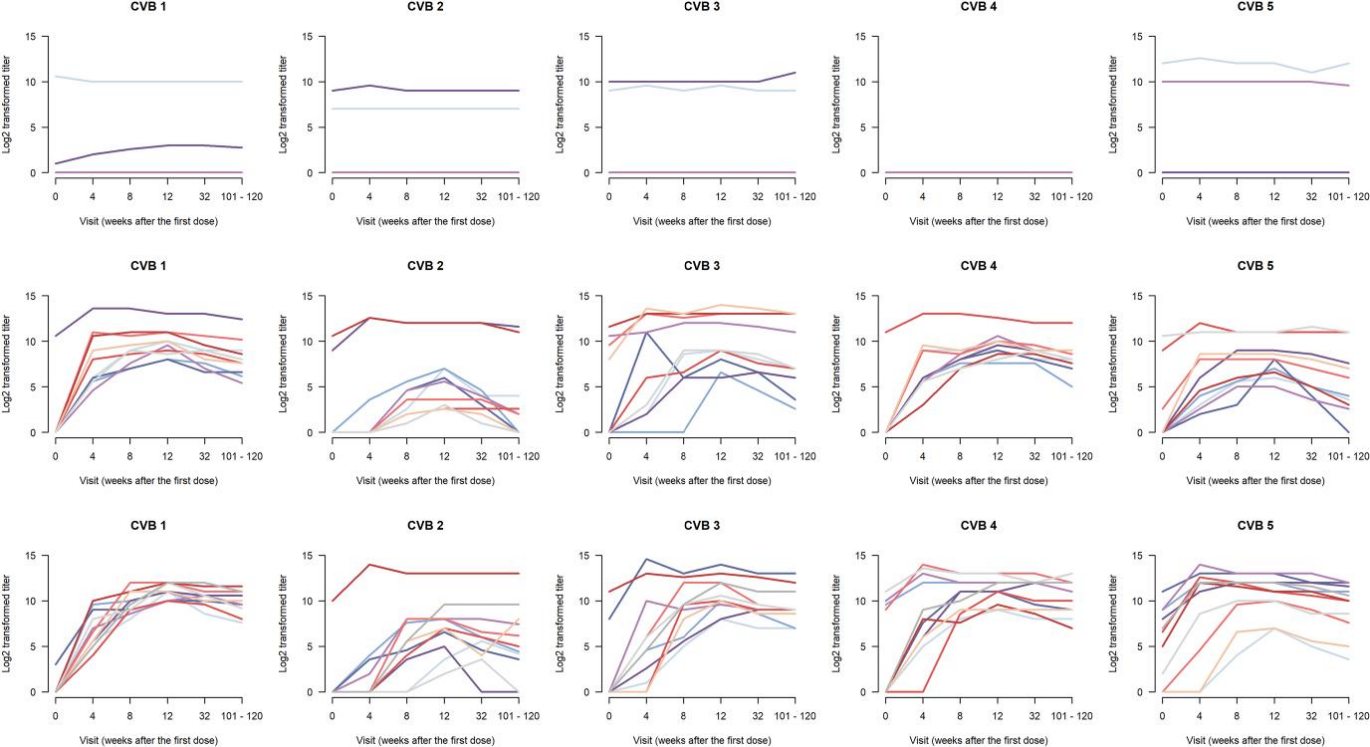
Neutralizing antibody titers (log scale) against CVB1–5 viruses in individual PROVENT-IIS trial participants. Each line represents 1 individual, its respective color staying the same through the virus antibody panels. The top panel represents the placebo group (n = 5), the middle panel the low-dose PRV-101 group (n = 10), and the bottom panel the high-dose group (n = 11). The IIS visit occurred 101–120 weeks after the first PRV-101 dose. The presumably protective titer 8 equals log2 value 3. All participants from the placebo group who were seronegative at baseline remained seronegative throughout follow-up (included in the horizontal line representing 0 in the top panel). Abbreviations: CVB, coxsackievirus B; IIS, investigator-initiated follow-up study.

**Figure 4.**
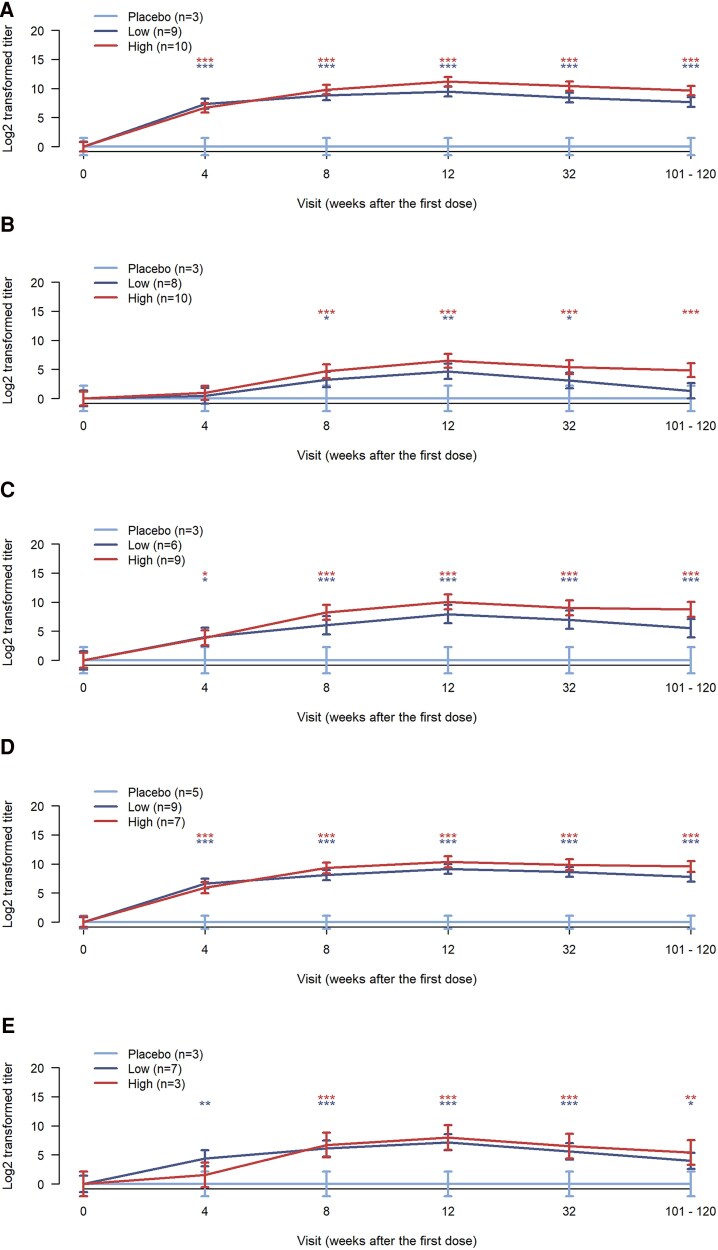
Neutralizing antibody titers (mean +/− SD calculated from log-transformed data) against CVB1–5 viruses in PROVENT-IIS trial participants who were initially seronegative at baseline for each tested CVB type (number of subjects shown in each panel). A, CVB1; B, CVB2; C, CVB3; D, CVB4; E, CVB5. The IIS visit took place 101–120 weeks after the first PRV-101 dose. The statistical significance of differences between the treatment and placebo arms is shown at each visit by asterisks (high-dose vs placebo = red asterisks; low-dose vs placebo = blue asterisks; *P < .05, **P < .01, and ***P < .001). Abbreviations: CVB, coxsackievirus B; IIS, investigator-initiated follow-up study.

An analysis of CVB neutralizing antibody titers over the whole trial period showed that PRV-101-induced neutralizing antibody responses persisted at the IIS time point (Figure 3; Supplementary Figures 1 and 2, Supplementary Table 1), remaining at elevated levels also in participants who were baseline seronegative (titer <4) for the tested CVB type (Figure 4; Supplementary Figures 3 and 5, Supplementary Table 2), including also those 5 participants who were baseline seronegative for all 5 CVB types (Supplementary Figure 4).

### Levels of the Neutralizing Antibody Titers in the High- and Low-Dose Groups

Among the baseline seronegative participants, clear dose dependency of the antibody response was observed. The higher dose of PRV-101 led to higher antibody levels at the IIS time point when compared with antibody levels in the lower-dose group. At the IIS time point, the difference between the high- and low-dose groups was statistically significant for all other CVB serotypes except CVB5 (Figure 4; Supplementary Figures 3 and 5, Supplementary Table 2). This difference was most obvious for CVB2, for which the vaccine induced generally weaker antibody responses compared with the other CVB components of PRV-101 (Supplementary Tables 1 and 2, Supplementary Figures 4 and 6). All baseline seronegative participants in the placebo group remained seronegative throughout follow-up.

In the high-dose group, all baseline-seronegative participants had IIS antibody titers of 4 or higher for CVB1, CVB3, CVB4, and CVB5, while CVB2 titers had declined below this level in 2 of the 10 baseline CVB2-seronegative subjects (Figure 3; Supplementary Figure 4). IIS titers remained at ≥4 for all CVB types in both of the 2 high-dose participants who were baseline seronegative for all 5 CVBs (Supplementary Figures 4 and 6).

In the low-dose group, all baseline-seronegative participants had IIS antibody titers ≥4 for CVB1, CVB3, and CVB4, while CVB2 titers had decreased <4 in 4 of the 8 such participants, and CVB5 antibodies had decreased <4 in 1 of the 7 such participants. All 3 low-dose participants who were baseline seronegative for all 5 CVB types had IIS titers of ≥4 against CVB1, CVB3, and CVB4, while this was the case for 1 of 3 participants initially seronegative for CVB2 and 2 out of 3 participants initially seronegative for CVB5 (Supplementary Figures 4 and 6).

As neutralizing antibody titers of ≥8 have been linked to protection against enterovirus infections (eg, regarding polioviruses) [8], we analyzed the proportion of baseline-seronegative participants whose titers remained at this level at the IIS time point. In the high-dose group, the results were identical with those for titers ≥4, whereas in the low-dose group, all baseline-seronegative participants had IIS antibody titers ≥8 for CVB1 and CVB4, while CVB2 titers had decreased <8 in 7 of 8 such individuals, CVB3 titers in 1 of 6, and CVB5 antibodies in 2 of 7 such individuals. In addition, the 3 low-dose participants who were seronegative at baseline for all 5 CVB types had IIS titers ≥8 against CVB1 and CVB4, while this was true for 1 of 3 such individuals for CVB2, 2 of 3 for CVB3, and 2 of 3 for CVB5 (Supplementary Figures 4 and 6).

### Coxsackievirus B Antibodies in the ELISA Assay

**Figure 5.**
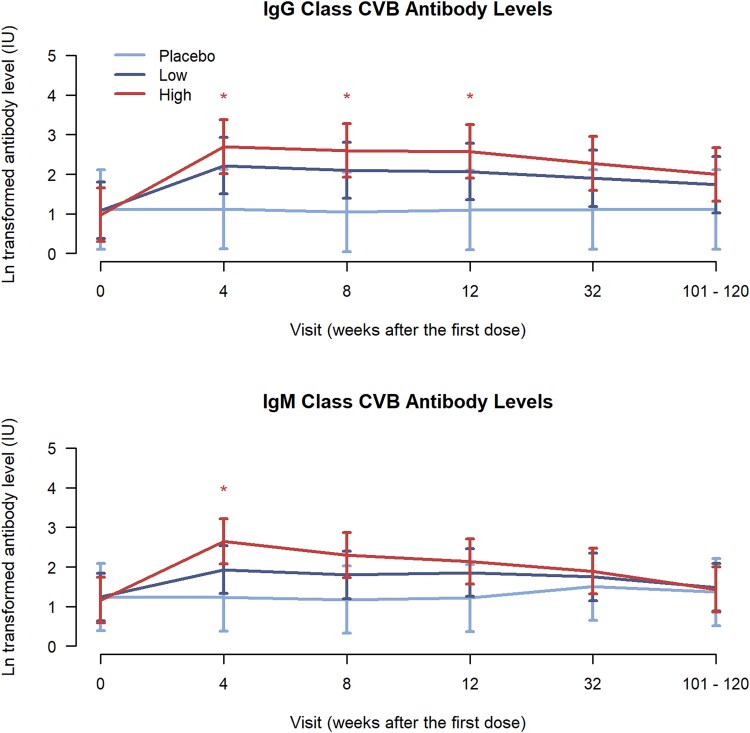
The levels of IgG and IgM antibodies (mean +/− SD calculated from log-transformed data) against CVB antigen in PROVENT-IIS trial participants, using Serion IgG and IgM ELISA assays. The statistical significance of differences between treatment and placebo arms is shown at each visit by asterisks (high-dose vs placebo = red asterisk; *P < .05). Abbreviations: CVB, coxsackievirus B; ELISA, enzyme-linked immunosorbent assay; Ig, immunoglobulin.

In ELISA assays, the levels of IgG class CVB antibodies peaked after the first vaccine dose and started to decrease at the 32-week time point (ie, 6 months after the third vaccine dose) both in the high– and low–PRV-101 dose groups (Figure 5; Supplementary Tables 1 and 2, Supplementary Figures 7–9). At the week 32 and IIS time points, IgG levels were still higher in the vaccinated groups than in the placebo group, but differences between the groups were no longer statistically significant. IgM antibodies decreased more steeply, declining to near baseline levels at the IIS time point. In the placebo group, no clear changes occurred in IgG or IgM antibody levels.

## DISCUSSION

The results of the present follow-up study indicate that PRV-101 can induce long-lasting antibody responses in initially CVB-seronegative individuals. Neutralizing CVB antibodies that correlate with protection against CVB infections remained elevated until ∼2 years after the last dose of PRV-101. Clear dose dependency was also seen—the participants in the high-dose group had higher neutralizing antibody levels, and they were more frequently seropositive (titer ≥4) at this late time point when compared with the participants in the low-dose group. In addition, in the high-dose group all baseline-seronegative participants reached presumably protective titers of ≥8 against CVB1, CVB3, CVB4, and CVB5, and 80% of them also for CVB2. Titers of ≥8 have been considered protective in previous poliovirus studies [8]. Similar dose-dependent patterns were also seen in IgG class CVB antibodies when measured with ELISA. Overall, these results suggest that 3 immunizations with the higher tested dose of PRV-101 led to robust and sustained immune responses, including the induction of neutralizing antibodies that mediate protection against CVB infections. The observation that PRV-101-induced antibodies remain elevated over such a long time suggests efficient induction of memory B cells by PRV-101 [9].

The results are well in line with the previous experience from other inactivated enterovirus vaccines. The widely used inactivated poliovirus vaccine (IPV) generates efficient protection against polio paralysis that lasts for decades [10]. In addition, several clinical trials and postlicensing studies have shown that similar formalin-inactivated vaccines against enterovirus 71 induce robust and long-lasting neutralizing antibody responses, providing good protection against enterovirus 71 infections [11]. Thus, it seems that formalin-inactivated whole-virus vaccines work well in immunization against several types of enteroviruses.

The antibody responses persisted also in the lower-dose group, but the response to CVB2, toward which the response was initially weaker than the responses against the other CVBs, waned in 7 of the 8 individuals who were CVB2 seronegative at baseline. Some individual variation in the waning of titers below titer 8 was also seen for CVB3 and CVB5 (in 1 of 6 individuals for CVB3 and in 2 of 7 individuals for CVB5, who were seronegative at baseline). As discussed in our previous paper [6], the lower concentration of CVB2 in the PRV-101 vaccine, compared with the other types, is the most likely explanation for the overall weaker responses to CVB2. In any case, the higher dose of PRV-101 seems to be a more favorable option for future clinical trials. However, it should be noted that the proportion of male participants was higher in the low-dose group than in the high-dose group. As males tend to mount weaker immune responses to vaccines than females, further studies are needed to confirm the observed dose–response patterns.

The finding that CVB antibodies remained elevated in immunized subjects who had been CVB seronegative at baseline, including those who had been seronegative for all CVBs, suggests that 3 immunizations with an appropriate dose of PRV-101 can induce long-term immunity in initially CVB-naïve individuals. However, even though neutralizing antibodies are considered reliable markers of past CVB infection, it is possible that some of these initially seronegative individuals had been previously exposed to CVBs and had preexisting CVB-specific memory T cells that can boost PRV-101-induced responses. Therefore, it would be important to carry out a similar trial among young children to confirm the high immunogenicity of PRV-101 in previously unexposed individuals. Young children are also the main target population of PRV-101 as it aims to prevent early CVB infections, which frequently cause severe diseases and can also contribute to the development of type 1 diabetes. The interval between the 3 immunizations was only 4 weeks, which was probably too short for optimally efficient immunization. Therefore, further studies are needed to test whether longer intervals could lead to stronger immune responses even with the lower-dose level of the vaccine. Such studies could also help to address the question of whether the response to CVB2 could be enhanced by modifying the dose or immunization protocol.

PRV-101 is manufactured using a similar technology as that used in the manufacture of IPVs (highly purified formalin-inactivated viruses). In addition, neither of these vaccines contains adjuvants. Vaccines against another enterovirus, enterovirus 71 (EV-71), have also been widely tested in phase 3 trials, showing excellent efficacy and safety, and one of these vaccines is licensed in China [11, 12]. These monovalent EV-71 vaccines have also been manufactured using formalin-inactivation technology similar to PRV-101. However, in contrast to PRV-101, they contain an alum adjuvant. Our preclinical studies in nonhuman primates showed that alum adjuvant did not augment the sustainability of neutralizing antibody response induced by a formalin-inactivated prototype vaccine containing all 6 CVB types [13]. Based on these facts, it seems that enterovirus vaccines that are based on formalin-inactivated whole-virus vaccine technology are efficient and safe in humans and can induce robust and sustained immune responses even without any adjuvant.

All IIS participants remained negative for all 4 type 1 diabetes–associated autoantibodies and celiac disease–associated transglutaminase autoantibodies. None of them had developed type 1 diabetes or celiac disease, or any other chronic illness. Thus, there was no indication that immunization with PRV-101 could increase the risk of developing autoimmune conditions. This is an important result for the future development of PRV-101, particularly as almost half of the IIS study participants carried HLA-conferred genetic susceptibility to type 1 diabetes or celiac disease [6]. Of note, PRV-101 does not contain any adjuvant or infective viruses. These are also important safety aspects of PRV-101 when considering immunizations among individuals who carry increased genetic risk to develop these or other autoimmune diseases. The safety of PRV-101 is also supported by the knowledge that the current inactivated poliovirus vaccine, which is produced using similar formalin-inactivation technology, is considered one of the safest vaccines ever developed. In any case, these favorable results, together with the relatively small number of participants in this phase 1 trial, argue for larger trials to get more detailed information about the tolerability, safety, and immunogenicity of PRV-101.

The Serion ELISA assays used to study the immunogenicity of PRV-101 are based on recombinant VP1 proteins of CVB1, CVB3, and CVB5 and are able to detect antibodies that cross-react between CVB serotypes and are therefore not specific to the individual CVB types that are used as antigen. The assays detected PRV-101-induced antibodies well, indicating that they are suitable for the evaluation of the immunogenicity of PRV-101 in human trials. In addition, VP1 protein seems to be an important target for PRV-101-induced antibodies.

In summary, the results of this investigator-initiated study indicate that immunization with PRV-101 was not associated with any late-appearing adverse effects during an observation period that lasted ∼2 years after the first administration of PRV-101. Furthermore, the CVB-neutralizing antibody responses that were induced by 3 immunizations with the higher dose of PRV-101 remained at high levels during this extended observation period, exceeding the presumed protective levels. Altogether, these findings support continuation of the PRV-101 clinical development program and make PRV-101 an attractive candidate for a vaccine that could prevent CVB-associated diseases and possibly also type 1 diabetes and celiac disease. Our next step is to confirm the safety and immunogenicity of PRV-101 in a Phase 1b trial in young children, who will be the main target group in clinical trials testing the efficacy of PRV-101 in the prevention of type 1 diabetes and celiac disease.

## Supplementary Material

ofag277_Supplementary_Data

## Supplementary Data

Supplementary materials are available at *Open Forum Infectious Diseases* online. Consisting of data provided by the authors to benefit the reader, the posted materials are not copyedited and are the sole responsibility of the authors, so questions or comments should be addressed to the corresponding author.
